# Histone H2A variants in nucleosomes and chromatin: more or less stable?

**DOI:** 10.1093/nar/gks865

**Published:** 2012-09-21

**Authors:** Clemens Bönisch, Sandra B. Hake

**Affiliations:** Department of Molecular Biology, Center for Integrated Protein Science Munich (CIPSM), Adolf-Butenandt-Institute, Ludwig-Maximilians-University Munich, 80336 Munich, Germany

## Abstract

In eukaryotes, DNA is organized together with histones and non-histone proteins into a highly complex nucleoprotein structure called chromatin, with the nucleosome as its monomeric subunit. Various interconnected mechanisms regulate DNA accessibility, including replacement of canonical histones with specialized histone variants. Histone variant incorporation can lead to profound chromatin structure alterations thereby influencing a multitude of biological processes ranging from transcriptional regulation to genome stability. Among core histones, the H2A family exhibits highest sequence divergence, resulting in the largest number of variants known. Strikingly, H2A variants differ mostly in their C-terminus, including the docking domain, strategically placed at the DNA entry/exit site and implicated in interactions with the (H3–H4)_2_-tetramer within the nucleosome and in the L1 loop, the interaction interface of H2A–H2B dimers. Moreover, the acidic patch, important for internucleosomal contacts and higher-order chromatin structure, is altered between different H2A variants. Consequently, H2A variant incorporation has the potential to strongly regulate DNA organization on several levels resulting in meaningful biological output. Here, we review experimental evidence pinpointing towards outstanding roles of these highly variable regions of H2A family members, docking domain, L1 loop and acidic patch, and close by discussing their influence on nucleosome and higher-order chromatin structure and stability.

## INTRODUCTION

In eukaryotes, DNA is organized into chromatin to fit into the constrained space of the nucleus. Generally, chromatin decreases the accessibility of DNA and consequently interferes with many biological processes, such as transcription, replication and repair, but helps to protect DNA from damage by different kinds of stress. Despite the immense degree of global compaction, access to DNA is achieved by local chromatin decondensation in a highly regulated manner. Chromatin is a dynamic structure allowing phenotypic plasticity. Its regulation involves several interconnected mechanisms (1), such as DNA methylation (2), adenosine triphosphate (ATP)-dependent chromatin remodelling (3), histone post-translational modifications (PTMs) (4), non-coding RNAs (ncRNAs) (5), arrangement within the 3D nuclear architecture (6) and the replacement of canonical histones by histone variants (7).

**Figure 1.**
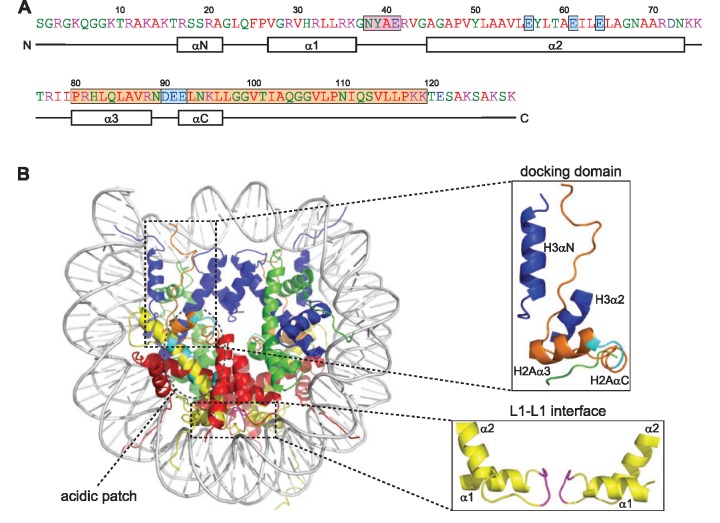
The crystal structure of the nucleosome. (**A**) Amino acid sequence of histone H2A type 1 from *Xenopus leavis* (NCBI reference sequence: NP_001089684.1). α-helices are indicated below and important structural features are highlighted with coloured boxes (L1 loop: magenta, acidic patch: cyan, docking domain: orange). The colour code for the amino acids is as follows: red: small, hydrophobic (A, V, F, P, M, I, L, W); blue: acidic (D, E); magenta: basic (R, K); green: hydroxyl, sulphydryl, amine, glycine (S, T, Y, H, C, N, G, Q). (**B**) Nucleosome crystal structure based on [(8), PDB ID: 1AOI]. H2A is shown in yellow, H2B in red, H3 in blue, H4 in green and DNA in light grey. L1 loop, acidic patch and docking domain are highlighted and shown in magenta, cyan and orange, respectively. Zoomed images of docking domain and L1–L1 interface are depicted on the right. All pictures were generated using PyMOL (14).

The ‘monomeric building block’ of chromatin, the nucleosome, contains ∼150 bp of DNA wrapped around a histone octamer consisting of two of each of the core histones H2A, H2B, H3 and H4 in 1.65 left-handed superhelical turns (8). The existence of a chromatin subunit, the nucleosome, was first proposed in 1973/74, based on regular patterns on nuclease digestion and electron microscopic analyses of chromatin [(9–11), for review see (12,13)]. About 25 years later, the nucleosome structure at 2.8 Å resolution revealed its fascinating details (8). The (H3–H4)_2_-tetramer is built by connecting two H3–H4 dimers at the dyad symmetry axis through a strong 4-helix bundle (4-HB) between the two H3 molecules. Interaction of H2A–H2B dimers with this tetramer is accomplished by a weaker 4-HB between H2B and H4. Additionally, interactions with H3 and H4 are provided by the C-terminal H2A docking domain that directs the H3 N-terminal helix to interact with DNA (Figure 1). Furthermore, contacts between the H2A L1 loops of the two H2A–H2B dimers stabilize their association within the nucleosome (Figure 1). However, the nucleosome is not a static entity but rather flexible and dynamic [see (12,13,15) and references therein]. As reviewed in van Holde and Zlatanova, evidence for nucleosomes organizing DNA lengths between 100 and 170 bp is abundant, stressing that the crystal structure with 147 bp must rather be viewed as a ‘snapshot’ of one possible state. In addition, recent single molecule analyses using Förster resonance energy transfer (FRET) contributed to the characterization of nucleosome dynamics, providing evidence for an alternative, more open nucleosome state (0.2–3% under physiological salt conditions *in vitro*) where all histones are bound to DNA, but the dimer/tetramer interactions are broken (12,16).

Histone variants are non-allelic isoforms of canonical histones that differ in their primary sequence and their expression timing (17). Expression of canonical histones is almost completely limited to S-phase, whereas most histone variants are expressed throughout the cell cycle. The S-phase dependent expression of canonical histones is mainly caused by their unique mRNA structure (18). In general, canonical histone genes lack introns, and their corresponding messenger RNAs (mRNAs) are not polyadenylated but have a unique 3′ stem loop crucial to modulate mRNA stability, transport and translation. In contrast, most histone variant mRNAs are polyadenylated, and their pre-mRNAs can contain introns (18). To date, only two histone transcripts have been shown to be alternatively spliced, macroH2A.1 (19) and H2A.Z.2 (20,21), giving rise to histone proteins with distinct functional and structural properties.

Histone variants contribute to chromatin complexity by creating specialized nucleosomes. Within nucleosomes, either one canonical H2A or both of them can be exchanged with a particular variant (heterotypic and homotypic nucleosomes, respectively), and such changes can have profound influences on nucleosome stability and biological outcome. Adding another layer of complexity, incorporation of variants of another histone family, for example, H3, can physically and functionally further diversify nucleosomes and thereby enhance chromatin complexity and plasticity, as we proposed several years ago (22). Hence, it is of utmost importance to decipher variant compositions of nucleosomes to understand the complex functional interplay of histone variants.

## THE HISTONE H2A FAMILY

**Figure 2.**
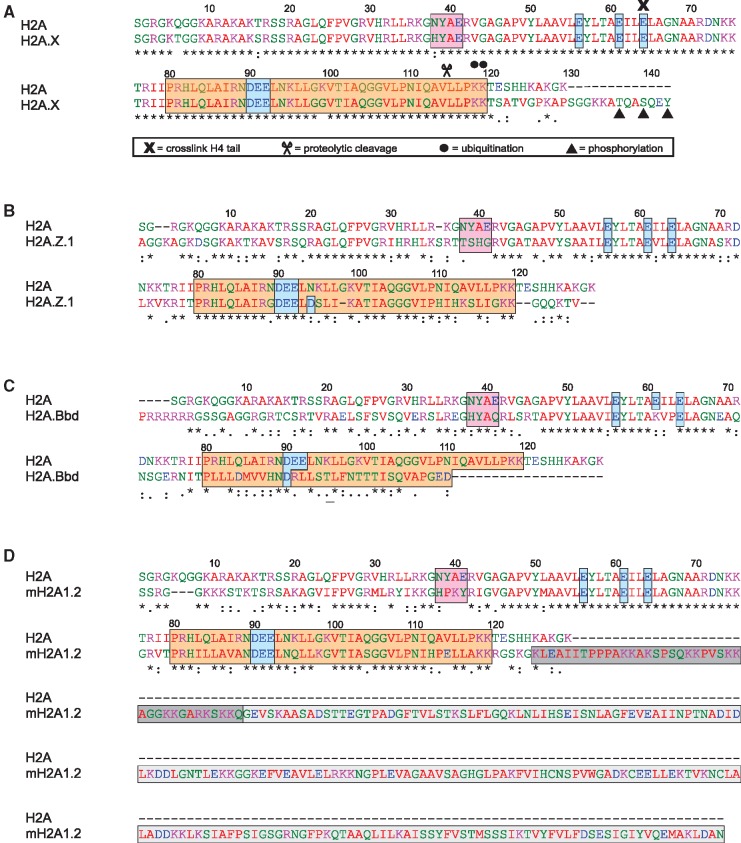
Amino acid sequences of human H2A variants. Alignments of human H2A type 1 (NCBI reference sequence: NP_003501.1) with human (**A**) H2A.X (NP_002096.1), (**B**) H2A.Z.1 (NP_002097.1), (**C**) H2A.Bbd (NP_001017990.1) and (**D**) macroH2A.1.2 (NP_004884.1). Important structural features are highlighted with coloured boxes. For details on colour coding see legend of Figure 1. H2A and H2A.X amino acids that are discussed in the text are highlighted according to the figure key. The consensus symbols below the alignment are as follows: an asterisk to indicate fully conserved residues, a colon to indicate conservation between groups of strongly similar properties and a period to indicate conservation between groups of weakly similar properties. (**D**) MacroH2A.1.2's linker region (amino acids 122–160) and macro domain (amino acids 161–370) are highlighted with dark grey and light grey boxes, respectively. All alignments were carried out using the ClustalW alignment tool on the EMBL-EBI homepage (28,29).

Because of the specific nucleosomal protein–protein and protein–DNA interactions of each of the core histones, they are subject to different degrees of structural constraint probably resulting in different potentials to evolve variants (7,23). For example, H4 is one of the most slowly evolving eukaryotic proteins (23), with variants only described in tetrahymena (24), trypanosomes (25) and the urochordate *Oikopleura dioica* (26). The H2A family, on the other hand, contains a plethora of variants with some ‘universal variants’ found in almost all organisms, namely H2A.Z and H2A.X (7). These different degrees of variation might be attributed to extensive intranucleosomal interactions in the case of H4 and the location of H2A on the ‘edges’ of the nucleosome. In general, the highest degree of diversification among histone H2A variants is to be found in their C-termini, regarding both length and amino acid sequence [(27) and Figure 2].

H2A’s C-terminus is located at the DNA entry/exit site (Figure 1), making variations at this domain a powerful tool to functionally diversify nucleosomes by altering nucleosome stability and dynamics, binding to DNA and/or the linker histone H1 or other interacting factors. Furthermore, the L1 region in the histone fold, where interaction between the two H2A variants takes place, shows a high degree of variation among H2A variants (Figure 2).

In addition to the *bona fide* H2A variants (discussed later in the text), canonical H2A proteins are not completely identical but rather show some sequence variability. In 1977, based on electrophoretic separation and subsequent analysis of amino acid composition, two different H2A isoforms (H2A.1 and H2A.2) that differ in amino acid position 51 (leucine or methionine, respectively) were identified in mammals (30). After the human and mouse genomes were sequenced, it became apparent that canonical H2A proteins can differ in many more positions, especially in the C-terminal six amino acids (17). However, thus far, no functional specialization of these canonical H2A isoforms has been demonstrated.

Interestingly, in addition to canonical H2A, H2A.X- and H2A.Z-like proteins, plants exhibit a special class of H2A isoforms that have an extended C-terminus comprising SPKK motifs (31,32) [according to the recently published unified phylogeny-based nomenclature for histone variants: H2A.W (33)]. This kind of motif (more general T/SPXK) is also present in many subtypes of the linker histone H1 and in sea urchin sperm-specific H2B, and it is a known target site for phosphorylation (34). This class of H2A proteins has been shown to protect ∼16 bp more linker DNA from micrococcal nuclease (MNase) digestion than chicken erythrocyte H2A (35). Their property to bind more DNA might help to compact the inactive genome during seed dormancy. Upon commencing germination, the H2A C-terminus is rapidly phosphorylated, probably to weaken DNA binding by neutralizing the positive charge of the SPKK motifs (36). A similar situation is found in the sea urchin egg-specific histone variant cleavage stage H2A. Here, C-terminal phosphorylation occurs upon fertilization, possibly leading to chromatin decondensation, which in turn could facilitate chromatin assembly during replication (34). Recently, the histones of bdelloid rotifers, freshwater invertebrates that are highly resistant to ionizing radiation and desiccation (37), were analysed (38). Interestingly, the H2A proteins in this organism are different from all other species with no canonical H2A, H2A.X or H2A.Z present. Instead, bdelloid H2As have longer C-termini that were speculated to play a role in adaption to their environment, especially dealing with DNA damage on desiccation (7,38).

In the next part, we briefly introduce the major H2A variants known thus far. For more detailed information about histone H2A variants and histone variants in general, see (7,39–41). The influence of H2A variants on nucleosome stability and on chromatin folding will not be addressed in this part but in separate sections later in the text.

### H2A.X

Histone H2A.X was, together with H2A.Z, first described in 1980 (42). H2A.X is defined by its SQ[E/D]Φ motif (where Φ is a hydrophobic amino acid) in the C-terminus. After DNA damage, this serine (position 139 in humans, see Figure 2A) becomes phosphorylated (γH2A.X) and renders H2A.X an important player in preserving genome integrity (see later in the text). Apart from the C-terminus, human H2A and H2A.X differ by just four amino acids in primary sequence; two substitutions in the N-terminal tail, (Q6T and T16S), one in the L1 loop (N38H) and one in the docking domain (K99G) (Figure 2A). However, as residue 38 is located in the region where the two H2A–H2B dimers interact with each other (Figure 1), it has been suggested that this substitution might influence the ratio of hetero- versus homotypic nucleosomes *in vivo* (43), which, to our knowledge, is not known. For a recent review on H2A.X structure and function, see (43). In some organisms, the SQ[E/D]Φ motif is present in other H2A family members; hence, no distinct H2A.X exists in these organisms. For example, in *Saccharomyces cerevisiae* and the flagellated protozoan *Giardia lamblia*, the SQ[E/D]Φ motif is part of the canonical H2A, whereas in the fly, a similar motif (SQAY) is present in the H2A.Z protein called H2Av [or H2AvD or D2, according to the unified phylogeny-based nomenclature for histone variants: H2A.Z.X to stress that it harbours characteristics of H2A.Z and H2A.X (33)]. Moreover, some organisms, such as bdelloid rotifers, *Caenorhabditis elegans* and some protists, lack H2A.X (43), indicating that its function is dispensable in some organisms.

During S-phase, the human and mouse H2A.X transcripts are processed in an identical manner to the canonical histone mRNAs, resulting in a stem loop structure and no polyA tail (44,45). Outside S-phase, a longer transcript is produced by using a downstream polyadenylation site. Therefore, *H2A.X* exhibits characteristics of both replication-dependent and replication-independent histone genes.

H2A.X has been shown to be involved in the DNA damage response (DDR). After DNA double strand break (DSB) occurence, H2A.X phosphorylation results in ‘γH2A.X foci’, which extend for up to 50 kb on each side of the DSB in *S. cerevisiae* (46) and for up to several Mb in mammals (47). H2A.X phosphorylation is an early event in DDR leading to structural alterations at the damaged site to foster DNA repair. Although there are studies pointing towards a direct destabilization of the nucleosome by γH2A.X (48,49), two studies in *S. cerevisiae*, using serine to glutamate mutants to mimic a phosphorylated serine, come to different results. Although one study (50) found increased nuclease accessibility, suggesting a more open chromatin structure, another one (51) did not find any evidence for a direct structural influence on chromatin. In contrast to these controversial reports, the importance of ATP-dependent chromatin remodelling during DDR is undisputed. Several studies established the crucial role of ATP-dependent chromatin remodelling complexes to increase DNA accessibility at the DSB site [reviewed in (52)]. As chromatin decondensation is not severely impaired in H2A.X knock-out cells (53,54), it has been suggested that the critical role of γH2A.X is not the primary recruitment of remodelling factors but their retention at the repair site to define a ‘damage neighbourhood’ and to keep the two DNA strands together for efficient repair [reviewed in (43)].

Moreover, γH2A.X and the DDR machinery are involved in the process of meiotic sex chromosome inactivation (MSCI) in mammals, the most prominent and best studied example of the more general process called meiotic silencing of unsynapsed chromatin (55). During meiosis, the homologous sister chromatids pair to allow homologous recombination. In male mammals, however, the X and Y sister chromatids pair only partially through their pseudoautosomal regions. The unpaired (unsynapsed) regions of the X and Y chromosomes (and unsynapsed regions of autosomes) become transcriptionally silenced and heterochromatinized, forming the XY body (or sex body) as a cytological entity (55). In this process, γH2A.X and its binding factor MDC1 (mediator of DNA damage checkpoint 1) play crucial roles, as in mouse, H2A.X (56) and MDC1 (57) null males, but not females, are infertile and do not undergo MSCI (58,59). Ichijima *et al.* (59) proposed a model in which initially induced γH2A.X spreads in an MDC1 dependent manner, resulting in chromosome-wide accumulation of γH2A.X and other DDR factors. Further, they propose that this leads to a downstream enrichment of repressive chromatin components through an as yet to be identified mechanism. Hence, as a key player during MSCI, the histone variant H2A.X influences chromatin structure, although indirectly, in a chromosome-wide scale.

Recently, two additional phosphorylation sites, in the vicinity of the extensively studied serine 139, have been reported (Figure 2A). Firstly, the very C-terminal tyrosine 142 in the SQEY motif can be phosphorylated in vertebrates (60–63). The modification status of this residue, which is absent in *S. cerevisiae* (L instead of Y), has been suggested to play a critical role in cell fate decision after DNA damage. If tyrosine 142 is phosphorylated, interaction of H2A.X with the pro-apoptotic c-Jun N-terminal kinase 1 is increased at the expense of DDR factor recruitment. Hence, its dephosphorylated form facilitates DNA repair, whereas its phosphorylated one promotes apoptosis. Secondly, phosphorylation of threonine 136 has been reported in mammals (49,64). Although the biological function of this modification is not yet known, it was speculated that together with serine 139 phosphorylation it might alter chromatin structure upon DNA damage (49).

Interestingly, a recent analysis of H2A variant dynamics in pre-implantation embryos suggested a novel role for H2A.X in chromatin remodelling during mouse development (65). The authors found a striking increase in H2A.X chromatin incorporation at the expense of canonical H2A, H2A.Z and macroH2A after fertilization, leading to chromatin containing mostly H2A.X and H2A during the one to four cell stages. Notably, this effect seems to depend primarily on H2A.X's C-terminus but not on serine 139, suggesting an intriguing and not well-understood effect of H2A.X on chromatin structure outside the DDR.

### H2A.Z

**Figure 3.**
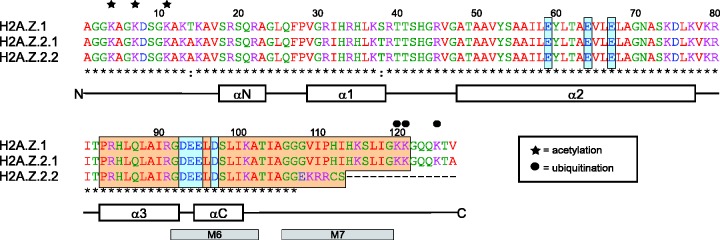
Amino acid sequences of human H2A.Z variants. Alignment of human H2A.Z.1 with H2A.Z.2.1 and H2A.Z.2.2. α-helices are indicated below and structural features that are discussed in the text are highlighted with coloured boxes. H2A.Z amino acids that are discussed in the text are highlighted according to the figure key. For details on colour coding and consensus symbols see legends of Figures 1 and 2, respectively. Sequence elements required for H2A.Z function (75) are indicated by grey boxes below and sites of PTMs as described in the figure key. Alignment was carried out using the ClustalW alignment tool on the EMBL-EBI homepage (28,29).

Histone H2A.Z is an almost universal variant, which evolved early and only once in evolution (66). H2A.Z is only ∼60% identical to canonical H2A within the same species [Figure 2B and (67)], but strikingly more conserved between different species (∼80% identity between most organisms), with the most divergent member in trypanosomes (∼50–60% identity) (68). These findings suggest that H2A.Z fulfils specific and unique functions that cannot be carried out by other H2A variants. Indeed, H2A.Z has been shown to be essential in many organisms like mouse (69), fly (70), frogs (71) and tetrahymena (72), but not in *S. cerevisiae* (73) and *Schizosaccharomyces pombe* (74), where knock-out leads to severe growth phenotypes. An elegant study in drosophila demonstrated that the essential regions for H2A.Z function are located in its C-terminus (M6 and M7, Figure 3) (75). In line with this finding, the M6 region is required for interaction of H2A.Z with the evolutionary conserved SWR-1 (Swi2/Snf2-related ATPase-1) chromatin remodelling complex important for H2A.Z targeting (discussed later in the text), providing a reasonable explanation for the essential nature of this region (76). Furthermore, M6 comprises residues of the acidic patch, important for H2A.Z deposition and function in *S. cerevisiae* (77), and chromatin higher-order structure (see respective section later in the text).

Surprisingly, despite significant sequence divergence, the H2A.Z nucleosome structure (78) revealed overall high similarity to the canonical one (8). Striking differences between both structures, however, are found in L1, important for interaction of the two H2A–H2B dimers within the nucleosome. Because of an expected sterical clash in heterotypic nucleosomes, these differences led to the hypothesis that the presence of canonical H2A and H2A.Z within the same nucleosome would strongly destabilize the particle; therefore, predicting the exclusive existence of homotypic H2A.Z nucleosomes (78). This prediction, however, was proven wrong *in vitro* (79) and *in vivo,* as heterotypic H2A.Z nucleosomes were found in *S. cerevisiae* (80), fly (81) and human (82). Further differences between the two structures were found in the C-terminal docking domain, suggesting a possibly altered interaction site for the linker histone or other factors, due to the presence of a metal ion at the nucleosomal surface. Additionally, an increased acidic patch on its surface is observed for the H2A.Z nucleosome (Figure 2), suggesting an influence on internucleosomal interactions [(78) and see later in the text]. Possible implications of the H2A.Z nucleosome structure for alterations of nucleosome stability, especially by L1 and docking domain, are discussed in the respective section later in the text.

Like other histones, H2A.Z can be post-translationally modified by acetylation, sumoylation and ubiquitination with different functional outcomes [Figure 3, reviewed in (83)]. H2A.Z sumoylation has been implicated in DNA repair in *S. cerevisiae* (84), ubiquitination correlates with localization to the inactive X chromosome (Xi) in mammals (85), whereas N-terminal acetylation leads to nucleosome destabilization (86). It was suggested that H2A.Z acetylation works as a switch-like mechanism to modulate H2A.Z nucleosome stability, ascribing repressive functions to the unmodified and activating functions to the acetylated form (87). Furthermore, acetylated H2A.Z was found associated with active genes, but its role at these sites is not yet completely understood [reviewed in (83)].

The biological function of H2A.Z has been extensively studied revealing roles in transcription regulation, DNA repair, heterochromatin formation, chromosome segregation and mitosis. Because of space constraints, we cannot discuss all aspects of H2A.Z biology. Excellent reviews covering the vast amount of literature are available (67,87–92).

Many studies focused on the influence of H2A.Z on transcription [reviewed in (87–89)], revealing that H2A.Z is enriched at gene promoters in *S. cerevisiae* (93–95), mammals (96) and plants (97). Interestingly, it has been found that H2A.Z can have both activating and repressive influences on transcription (87). Evidence accumulated that H2A.Z affects nucleosome mobility and positioning (87,94,98–102), which could explain the sometimes rather contrasting impact on transcription. As a consequence of such changes, incorporation of H2A.Z could differentially increase or decrease binding of activating and repressive regulatory factors to their target sequences. Hence, the naïve view of H2A.Z as a transcriptional activator (or repressor), acting merely by structural alterations, should be extended by one interpreting H2A.Z as a modulator of nucleosome positioning, which consequently influences different biological processes, including gene activity by transcription regulation. In addition to gene promoters, H2A.Z is associated with other regulatory regions like enhancers and insulators as well as heterochromatin [reviewed in (67)], consistent with the wide variety of biological processes this variant is implicated in. Moreover, in plants, H2A.Z and DNA methylation localize mutually exclusively, thereby providing a plausible mechanism for transcriptional regulation by these two important chromatin marks, with H2A.Z having a positive and DNA methylation having a negative effect on transcription (97).

The intriguing finding that H2A.Z is non-uniformly localized within the genome leads to the question by which means H2A.Z is enriched at its target sites. Three non-mutually exclusive mechanisms can be envisioned. Firstly, H2A.Z can be actively incorporated at specific sites by targeting factors; secondly, H2A.Z can be randomly incorporated and afterwards (actively) removed from non-target sites (103) and thirdly, H2A.Z localization can be explained by differential stabilities of homotypic H2A.Z nucleosomes compared with heterotypic or canonical ones (81) [for recent reviews on H2A.Z deposition see (90,91,103)].

In support of the first model, the ATP-dependent chromatin remodelling complex SWR-1 is important for H2A.Z deposition in *S. cerevisiae* by exchanging nucleosomal H2A–H2B dimers (H2A–H2B) for free H2A.Z–H2B dimers (H2A.Z–H2B) in a stepwise manner (80,104–106). Here, target sites for H2A.Z incorporation can be defined in at least two different ways. On the one hand, it was suggested that the SWR-1 complex can be recruited by acetylated histones (93,95); on the other hand, the insertion of a certain DNA sequence that harbours common *S. cerevisiae* promoter elements into an inactive gene was shown to be sufficient to induce a typical feature of *S. cerevisiae* promoters: a nucleosome-free region (NFR) flanked by two H2A.Z-containing nucleosomes (95). Therefore, both DNA sequence and chromatin modifications contribute to establish the specific H2A.Z pattern in this organism. Importantly, as SWR-1 catalyses the eviction of H2A–H2B and the insertion of H2A.Z–H2B, it might, in the absence of H2A.Z, only evict H2A–H2B without inserting H2A.Z–H2B. Hence, in an H2A.Z knock-out background, the presence of SWR-1 might be detrimental and partially responsible for the observed phenotypes. Accordingly, knock-outs of SWR-1 components that disrupt complex assembly or function suppress H2A.Z knock-out phenotypes at least partially (107,108). In mammals, the highly conserved SWR-1 complex has two related counterparts, p400/NuA4/TIP60 (E1A-binding protein p400/Nucleosomal Acetytransferase of H4/Tat-Interactive Protein 60) and SRCAP (Snf2-Related CREBBP Activator Protein) [(92) and references therein]. Both complexes can catalyse the exchange of nucleosomal H2A–H2B for free H2A.Z–H2B, but their different compositions implicate functional specialization.

In support of the second model, more recently, the importance of the ATP-dependent chromatin remodelling complex INO80 (Inositol-requiring protein 80) for H2A.Z localization patterns was established in *S. cerevisiae* (109). Papamichos-Chronakis *et al.* found that INO80 catalyses the opposite reaction as SWR-1, namely the active exchange of nucleosomal H2A.Z–H2B for free H2A–H2B. It is important to note that another study did not find data supporting this activity of the INO80 complex (80); however, this could be explained by an insufficiently low concentration of canonical nucleosomes (∼15 nM), as a concentration of >50 nM is required for full stimulation of INO80's histone exchange activity (109). Addressing the *in vivo* relevance of this reaction, Papamichos-Chronakis *et al.* found that loss of INO80 leads to mislocalization of unacetylated H2A.Z concomitant with genome instability. Speculating about the underlying mechanism, they hypothesized that impairment of removal of unacetylated H2A.Z might interfere with processes ensuring genome stability by altering chromatin structure.

The third model was put forward by Weber *et al.* (81) to explain their finding that only homotypic H2A.Z nucleosomes are enriched over genes in the fly. Under the assumption that homotypic H2A.Z nucleosomes are more stable than heterotypic or canonical ones, which could be envisioned because of the different interfaces between the two H2A–H2B dimers within the nucleosome (L1) or more stable interactions of the H2A.Z docking domain with H3 (see next section for discussion of H2A.Z nucleosome stability), they proposed that unique intranucleosomal interactions could be crucial determinants for homotypic H2A.Z nucleosome localization. In their model, RNA polymerase II transit leads to loss of an H2A–H2B dimer from the nucleosome during transcription, which can be replaced by either H2A–H2B or H2A.Z–H2B. Because of their enhanced stability, homotypic H2A.Z nucleosomes are disrupted less often than heterotypic or canonical ones, and hence, become enriched after several rounds of transcription. Interestingly, they reported that heterotypic H2A.Z nucleosomes are uniformly distributed throughout the genome, which might reflect the H2A.X function in DDR of fly H2A.Z (H2Av), the only H2A variant present in this organism, thereby ascribing different functions to a histone variant dependent on homotypic or heterotypic nucleosome composition.

In vertebrates, two non-allelic H2A.Z genes exist, *H2A.Z.1* (*H2AFZ*) and *H2A.Z.2* (*H2AFV*), which are expressed in a wide variety of tissues (20,110). Both genes contain introns, give rise to polyadenylated mRNAs and to protein products that differ in only three amino acids [Figure 3 and (111)]. Both H2A.Z proteins can be acetylated at the same N-terminal lysine residues [Figure 3 and (110)], and they show similar nuclear localization patterns (20,110) and fluorescence recovery after photobleaching (FRAP) mobilities (20). Their promoter structures, however, are different between both genes (110), and knock-out of *H2A.Z.2* but not of *H2A.Z.1* leads to BCL6 downregulation and increased apoptosis in chicken DT40 cells, suggesting functional (sub)specialization of the two H2A.Z variants (112).

Recently, we (20) and others (21) showed that the human H2A.Z.2 transcript can be alternatively spliced giving rise to two isoforms, the already known H2A.Z.2.1 (Z.2.1, formerly H2A.Z-2) and the novel H2A.Z.2.2 (Z.2.2). In contrast to the highly conserved major isoform Z.2.1, Z.2.2 is putatively primate-specific and present at much lower levels in most tissues. In brain tissues, however, Z.2.2 is significantly enriched with abundances similar to Z.2.1. The two alternatively spliced transcripts differ only in their last exons, resulting in differences only in the C-termini of the encoded proteins. Z.2.2 is the shorter protein, lacking the utmost C-terminal tail and having a unique C-terminus/docking domain but retaining the extended H2A.Z acidic patch completely (Figure 3). Furthermore, we could show that Z.2.2 nucleosomes exhibit striking differences with regards to nucleosome stability *in vivo* and *in vitro* [(20) and discussed in the respective section later in the text].

Notably, it has been suggested that alternative splicing can be a significant evolutionary driving force, since alternative splicing events are often, as in the case of H2A.Z.2, associated with exon gain or loss when compared between human, mouse and rat (113,114). Modrek and Lee (113) proposed that alternatively spliced isoforms can serve as ‘internal paralogues’. Initially underrepresented because of weak splice signals, they can be tolerated, as they do not interfere with gene function and are not detrimental for the cell. Over time, however, they are able to accumulate mutations and become functionally important in a tissue-specific manner, where they can represent 30–70% of all transcript isoforms from the respective locus (113). Indeed, >90% of human multi-exon genes are alternatively spliced with splicing patterns varying between different tissues (115). These findings are in perfect agreement with data from us (20) and others (21), showing that Z.2.2 mRNA is normally low abundant but constitutes up to 50% of the H2A.Z.2 isoforms in brain tissues. It is tempting to speculate that Z.2.2 is the major H2A.Z.2 isoform in some specialized cell types in the primate brain. There, it might be able to substitute for Z.2.1 and to confer unique structural and functional properties to nucleosomes, possibly acting in concert with chaperone complexes containing brain specific subunits (116,117).

### H2A.Bbd

Histone H2A.Bbd (Barr body deficient) was first described over one decade ago (118), but identification of the endogenous protein from mouse was published only recently (119). As found most often for replacement variants (17), H2A.Bbd is encoded by a polyadenylated mRNA. On the protein level, H2A.Bbd is only ∼50% identical to canonical H2A [Figure 2C and (118)] and is the most quickly evolving histone variant known, even exceeding the rate of evolution of the linker histone H1 (119,120). In agreement with its fast evolution, several H2A.Bbd-like proteins [also known as H2AL1-3 (121) or H2A.Lap2-4 (122)] are found in mouse, which are not all present in the human genome (121). Thus far, H2A.Bbd has only been found in mammals (120).

Comparison of histone H2A.Bbd and H2A protein sequences reveals several striking differences (Figure 2C). H2A.Bbd is considerably shorter, lacking the C-terminal tail and part of the docking domain. Additionally, it does not contain an acidic patch implicated in internucleosomal contacts and chromatin fibre condensation (see later in the text). Therefore, H2A.Bbd is also called H2A.Lap1 (lack of acidic patch) in mouse (122). We decided to stick to the more widely used term H2A.Bbd in the following text. However, a novel nomenclature for histone variants suggests using H2A.B instead of H2A.Bbd or H2A.Lap1, and H2A.L.1 instead of H2AL1, respectively (33). Interestingly, H2A.Bbd contains relatively few lysine residues, indicating poor conservation of possible modifications, for example, acetylation in the N-terminus.

H2A.Bbd is not present in all tissues but is strongly expressed in testis (118,119,122) and to a much lesser extent in brain (122), suggesting a tissue-specific function. Indeed, H2A.Bbd plays a role in mouse spermatogenesis (119,122). Soboleva *et al.* could show that H2A.Bbd is involved in creating a specific chromatin landscape at the promoters of active genes, during spermatogenesis, in a temporally specific manner, where H2A.Z occupies the −2 nucleosome and H2A.Bbd the −1 nucleosome with respect to the transcription start site (TSS) of active genes. As incorporation of H2A.Bbd hinders chromatin fibre folding to a similar extent like acetylated H3 and H4, the authors suggested that using H2A.Bbd instead of histone acetylation could be advantageous in the process of rapid chromatin remodelling during spermatogenesis in mouse. H2A.Bbd's association with actively transcribed chromatin is further supported by co-localization of ectopically expressed H2A.Bbd with acetylated H4 (118).

Recently, Tolstorukov *et al.* (123) investigated human H2A.Bbd's biological function in HeLa cells. In accordance with the studies discussed earlier in the text, they found that H2A.Bbd associates with active genes. However, in contrast to endogenous mouse H2A.Bbd/H2A.Lap1 (see earlier in the text), ectopically expressed, tagged human H2A.Bbd is depleted at the TSS but enriched over gene bodies. Consistent with its genome-wide localization, identification of proteins associated with H2A.Bbd chromatin pointed towards an enrichment of factors involved in transcription and mRNA processing. In addition to analyses involving tagged H2A.Bbd, the authors also examined transcriptome changes after depletion of H2A.Bbd or H2A.Z. Interestingly, knock-down of either of the two variants leads to reduced efficiency of mRNA splicing, with H2A.Bbd knock-down showing significantly stronger effects, therefore, implicating histone variants in mRNA processing. Although consistent with the experiments using tagged H2A variants, knock-down experiments of endogenous H2A.Bbd in HeLa cells can be problematic and can lead to artifacts because of its very low abundance in this cell line (unpublished own data), which is, according to the authors, not detectable by western blot (123).

To address the mechanism by which H2A.Bbd influences transcription, experiments *in vitro* have been employed by several groups. Surprisingly, different studies came to different results. Although two studies reported only mild effects on transcription of an H2A.Bbd chromatin array compared with a canonical H2A one (124,125), another study found transcription to be ∼5-fold more efficient on an H2A.Bbd template (126), consistent with the *in vivo* results discussed earlier in the text. Moreover, transcription efficiency dependence on the acetyltransferase p300 was different in different studies. Although one study (124) found a more pronounced increase in transcription efficiency from H2A.Bbd than from canonical H2A arrays, because of the presence of p300 and coincident with elevated histone acetylation, another one (125) reported that p300 activity levels out expression from both kinds of templates. In addition to analysing transcription efficiency, Angelov *et al.* found a maximal 2-fold increased interaction of a transcription factor (NF-κB) with its binding site for H2A.Bbd versus canonical H2A nucleosomes *in vitro*.

Interestingly, and counter-intuitively, H2A.Bbd is much less efficiently remodelled by a variety of ATP-dependent chromatin remodelling complexes like SWI/SNF (SWItch/Sucrose Non-Fermentable), ACF (ATP-utilizing Chromatin assembly and remodelling Factor) (124) and RSC (Remodels the Structure of Chromatin) (127). However, as H2A.Bbd is expressed more or less testis-specifically, it is possible that it is remodelled by, as yet, unidentified, tissue-specific, molecular machines.

In general, testis-specific variants of other histone families, such as H3t (128) and hTSH2B (129), are also known [TS H3.4 and TS H2B.1, respectively (33)]. Together, they contribute to the unique chromatin structure in testis and are speculated to be involved in the process of histone to protamine replacement. However, because of the tissue-specific expression of H2A.Bbd and other testis-specific histone variants, studies are limited in the generality of their implications on chromatin structure in other tissues. For most tissues and cells types, chromatin structure cannot be influenced by variants like H2A.Bbd simply because of their absence or low expression levels (Z.2.2). Hence, structural alterations must be accomplished by other, more general (or as yet unknown tissue-specific) means. On the other hand, tissue and cell type-specific histone variants (and other chromatin factors) could contribute to specialized chromatin functions only required in certain cell types and tissues like testis (H2A.Bbd) and brain (Z.2.2).

### MacroH2A

MacroH2A was first described two decades ago (130) and has since fascinated researchers because of its particular domain architecture. It has a tripartite structure consisting of an N-terminal histone domain connected through a lysine rich H1-like linker region to a non-histone macro domain (Figure 2D), resulting in a protein about three times larger than canonical H2A. The highly conserved macro domain is a binding module for nicotinamide adenine dinucleotide (NAD) metabolites and implicated in diverse biological functions like transcriptional regulation, chromatin remodelling and DNA repair [for a recent review on macro domain proteins see (131)]. MacroH2A is found in many animals, invertebrates and vertebrates (7), whereas macro domain-containing non-histone proteins are found in all organisms (132). Two macroH2A genes are present in vertebrates (*macroH2A1/H2AFY* and *macroH2A2/H2AFY2*) (7), with one of them (*macroH2A1*), known to be alternatively spliced (19), giving rise to two isoforms, macroH2A.1.1 and macroH2A.1.2. The two splice variants differ only in their macro domains, resulting in differences in their abilities to interact with NAD metabolites (133,134). MacroH2A.1.1 can bind NAD metabolites, including poly(ADP-ribose), whereas macroH2A.1.2 cannot. This suggests a unique role for macroH2A.1.1 in chromatin remodelling that depends on poly-ADP-ribose polymerase (PARP) activity, which is induced by different biological stimuli, such as DNA damage and metabolic stress.

The first insights into macroH2A's biological function(s) came from immunofluorescence microscopy studies showing an enrichment on the Xi in female mammals (135). Mammalian dosage compensation is accomplished by transcriptional silencing of one of the two X chromosomes in females resulting in the same gene dose as in males [recently reviewed in (136)]. The Xi is a *bona fide* model for an epigenetically regulated chromatin state, as, once established, it is stably passed on during mitosis. These initial findings constituted the basis for the general view of macroH2A as an epigenetic repressor of gene transcription involved in X inactivation. Recently, nuclear transfer experiments in frogs showed that macroH2A inhibits reprogramming and, hence, contributes to stability and maintenance of differentiated epigenomes (137). Although macroH2A's role in X inactivation is well established, two findings suggested early on function(s) outside X inactivation. MacroH2A is also present in other vertebrates that do not undergo X inactivation, and it is expressed equally in male and female mammals (19,132).

Many studies analysed the influence of macroH2A on gene expression on the X chromosome and autosomes. The general view is that macroH2A represses transcription by setting up a repressive chromatin environment [(135,138–140) reviewed in (132,141)]. However, some recent studies challenge this view by reporting a positive influence on some macroH2A target genes, thereby also influencing cell differentiation (142–144). How macroH2A mechanistically works on these target genes is not well understood.

In 2005, the structure of the macroH2A-containing nucleosome was published showing overall similarity when compared with the canonical one (145). The structure of the macroH2A docking domain, although harbouring several substitutions, is not altered, and the residues constituting the acidic patch are completely conserved. However, the two structures differ substantially in a four amino acid region in the L1 loop, which constitutes the interaction site of the two H2A–H2B dimers within the nucleosome. Interestingly, macroH2A preferentially forms heterotypic nucleosomes over homotypic ones *in vitro* with an overall similar structure but changes in the L1–L1 interface due to the amino acid sequence differences in the two H2A variants (146). This is in contrast to H2A.Z and H2A.Bbd, which form stochastic mixtures of homo- and heterotypic nucleosomes *in vitro* (79). In addition to affecting nucleosome structure, the L1 region of macroH2A might play a role in Xi targeting, because, when inserted into canonical H2A, it is sufficient for Xi enrichment (147). Together with two other regions sufficient for Xi targeting (one in the α1-helix and one in the docking domain), the L1 region is located on the outside of the macroH2A–H2B dimer, constituting a possible chaperone-binding site (147).

In contrast to H2A.Z, factors involved in macroH2A targeting are not well characterized; only one recent study provides first insights (148). Ratnakumar *et al.* showed that macroH2A associates with ATRX (α-Thalassemia/Mental Retardation syndrome X-linked), although it is not known whether this interaction is direct or not. Importantly, macroH2A–ATRX interaction is independent of DAXX (Death-Associated protein 6), which acts together with ATRX in H3.3 deposition at telomeres (149–151). These findings demonstrate that two distinct ATRX-containing complexes act together on H3.3 and macroH2A. Interestingly, in contrast to its role in active H3.3 deposition (149,150), ATRX is a negative regulator of macroH2A chromatin association by an, as yet, unknown mechanism. ATRX knock-down leads to increased macroH2A incorporation at telomeres and the α-globin gene cluster, concomitant with its reduced expression. Together with the study by Papamichos-Chronakis *et al.* (see earlier in the text), this one contributes to the emerging view of the thus far underappreciated importance of regulation of histone variant localization by factors negatively influencing their chromatin association.

Several studies used *in vitro* experiments to gain insight into the mechanisms by which macroH2A functions to repress transcription. Angelov *et al.* (152) suggested that macroH2A acts on at least two distinct levels to repress transcription. Firstly, by interfering with transcription factor binding if the binding site is close to the nucleosome dyad axis, the part which shows strongest alteration in DNaseI digestion pattern and, secondly, by inhibiting ATP-dependent nucleosome remodelling. The authors found that the influence on transcription factor binding is dependent on macroH2A's non-histone region (NHR; linker and macro domain, amino acids 121–372), whereas the histone domain alone is sufficient to inhibit remodelling. Another study, however, reinvestigated nucleosome remodelling of macroH2A with different results (153). Here, ATP-dependent nucleosome remodelling of macroH2A nucleosomes by SWI/SNF and ACF was not found to be impaired. Using competition experiments, they could show that the activating SWI/SNF complex binds preferentially canonical over macroH2A nucleosomes, whereas the ACF complex, mostly involved in gene repression, does not show any preference. Interestingly, all effects were found to be dependent on the NHR in contrast to the study by Angelov *et al.*

In addition to the mechanisms discussed earlier in the text, macroH2A also represses transcription more indirectly by reducing histone acetylation through different mechanisms dependent on the NHR. On the one hand, macroH2A inhibits p300-dependent histone acetylation *in vitro* (154); on the other hand, it interacts with lysine deacetylases and co-precipitates with hypoacetylated chromatin (145).

Taken together, the H2A family has a multitude of different members that differ strikingly with regards to their evolutionary conservation, amino acid sequences and domain architectures, and the biological processes they play roles in. The mechanisms of their functions are often not well understood; open questions remain, including how they are targeted to their respective chromatin sites, and how specific interaction partners contribute to their biological roles. One plausible mechanism of function is the alteration of nucleosome and higher-order chromatin structure brought about by H2A variant incorporation. In the next section, H2A variants and the properties they confer to chromatin on different levels, ranging from the nucleosome to higher-order chromatin structure, are discussed.

## THE INFLUENCE OF H2A VARIANTS ON NUCLEOSOME STABILITY

### The H2A C-terminus influences nucleosome properties, such as stability, dynamics, positioning and linker histone binding

Core histones have a common structural architecture, as they consist of a histone fold domain (three α-helices connected by short loops) and an unstructured N-terminal tail (8). In addition and in contrast to the other core histones, H2A also exhibits a flexible tail at the C-terminus. From the crystal structure, it can be seen that the C-terminal part of H2A (amino acids 80–119), including the α3- and αC-helices, forms a ladle shaped docking domain that constitutes an important interface for interaction with the (H3–H4)_2_-tetramer (8). The very C-terminal amino acids protrude from the globular nucleosome structure and interact with DNA, which is illustrated by molecular dynamics (MD) simulations, revealing stable hydrogen bonds between DNA and the lysines 118 and 119 in H2A (155). This is consistent with the recent finding that H2A monoubiquitination of these residues (Figure 2A) destabilizes nucleosomes during repair of UV-induced DNA damage (156), possibly by neutralization of the negative charge of the ε-amino group. Interactions of the H2A C-terminus with nucleosomal DNA are modulated by the presence of linker DNA and the linker histone H1 (157,158). Moreover, H2A can directly interact with H1, as has been shown by crosslinking experiments (159,160) and, recently, this interaction site was mapped to the last 17 amino acids of H2A, further stressing the importance of its C-terminal tail (161).

### H2A variants affect linker histone binding

The question of whether linker histone binding to nucleosomes is affected by H2A variant incorporation has been addressed for all major H2A variants *in vitro*. In general, canonical H2A nucleosomes seem to bind the linker histone most efficiently, in accordance with a direct interaction between the two proteins (159–161). Incorporation of H2A.X into nucleosomes exhibits only mild effects on interaction with H1, but phosphorylation of the H2A.X C-terminus leads to significant impairment of this binding (49). More pronounced reductions of interaction with H1 were reported for H2A.Z (102) and H2A.Bbd nucleosomes (127). In the case of H2A.Bbd, this has been further dissected, showing that reduced interaction with H1 can be attributed to the H2A.Bbd docking domain. In contrast to the other variants, the influence of macroH2A on H1 binding has not been analysed using *in vitro* assembled nucleosomes. However, fractionation experiments of native chicken chromatin revealed an almost mutually exclusive distribution; chromatin is either associated with linker histone or it contains macroH2A (162). This finding suggests that macroH2A interferes with linker histone binding, probably by its large C-terminal NHR, but it does not address the question of whether macroH2A incorporation influences H1 binding as directly as the band shift assays, which were carried out for other H2A variants (see earlier in the text). Notably, it has been shown that the H1-like linker domain of macroH2A decreases accessibility of extranucleosomal DNA at the entry/exit site of the nucleosome (163) and fosters chromatin folding and compaction (164), leading to the notion that this particular domain might fulfil linker histone function. Importantly, these effects were only found in the absence of the macro domain, possibly being functionally relevant after removal of the macro domain by proteolytic cleavage (164). However, no evidence pointing towards any occurrence of this mechanism *in vivo* is available thus far.

### Interactions of the H2A C-terminus within the nucleosome define nucleosomal properties

The importance of the H2A C-terminus for protein–protein interactions within the histone octamer was established ∼25 years ago (165). Eickbush *et al.* found that removal of H2A's 15 C-terminal amino acids resulted in a significant destabilization of the isolated histone octamer under high-salt conditions; the histone octamer is unstable under physiological salt conditions but can be stabilized by high-salt concentrations (166). Cleaving the peptide bond between valine 114 and leucine 115 (Figure 2A) destroys a short α-helix (Q112–L116) that is present in the isolated octamer (167) as well as the nucleosome (168) and contributes to complex stability by hydrophobic interactions between H2A and H3 (167). Recently, Vogler *et al.* (161) analysed C-terminal truncations of canonical H2A *in vitro* and *in vivo*. They reported moderately decreased nucleosome stability because of removal of the C-terminal 15 amino acids (Figure 2A). More interestingly, they also found altered nucleosome positioning and less H1 binding as well as decreased susceptibility to ATP-dependent chromatin remodelling, consistent with data from others (127). The biological significance of these findings is illustrated by reduced stress resistance of cells expressing H2A truncations at levels of ∼10% of endogenous H2A, probably brought about by altered chromatin structure because of insufficient H1 recruitment and erroneous nucleosome positioning. Furthermore, C-terminal truncations of H2A enhance thermal nucleosome mobility, pointing towards the influence of the H2A C-terminus in defining specific and stable nucleosome positions (161,169). Analysis of H2A N-terminal truncations *in vitro* showed only a mild increase in thermal mobility but changes in nucleosome positioning (169), consistent with interactions of the H2A N-terminus with the nucleosome core (170). The possible role of H2A.Z in creating nucleosomes harbouring special properties with regards to nucleosome positioning and mobility (see earlier in the text), further highlights the importance of H2A variants in defining unique nucleosomal properties, probably by their unique N- and C-terminal histone tails.

The evidence for an alternative nucleosome state in which all histones are bound to DNA but where the interface between the (H3–H4)_2_-tetramer and the H2A–H2B dimer is opened, suggests an intriguing model for the influence of H2A variants on nucleosome stability and dynamics (12,16). H2A variant incorporation can lead to alterations of this particular interface thereby shifting the equilibrium between the closed and the open nucleosome state and consequently confers distinct dynamic properties to variant-containing nucleosomes. As H2A variants differ significantly in their C-termini that are implicated in these interactions, this could be one mechanism by which they accomplish their distinct biological functions. Consistent with the idea that the H2A–H3 interface is sensitive to changes on both sides and critical for nucleosome properties, mutations of residues within the H3 αN-helix (I51A or Q55A), involved in interactions with the H2A C-terminus, greatly increase nucleosome thermal mobility, H2A–H2B dimer exchange and abolish octamer formation under high-salt conditions *in vitro* (169). The influence of the different H2A variants on nucleosome stability is discussed later in the text.

### H2A.X

In general, the biochemical and biophysical properties of H2A.X have not been studied as extensively as for the other major H2A variants. This might be due to its high similarity to canonical H2A. However, a recent study analysed the stability of H2A.X- and γH2A.X-containing nucleosomes by analytical ultracentrifugation (49). Surprisingly, the authors found striking nucleosome destabilization by H2A.X, which was further enhanced by C-terminal phosphorylation (Figure 2A). Indeed, human H2A.X harbours two substitutions in comparison with H2A (N38H and K99G) that were suggested to influence nucleosome stability, as they are located in regions important for protein–protein interactions within the nucleosome (43). Li *et al.* state that the observed destabilization of H2A.X nucleosomes is similar to observations of *S. cerevisiae* nucleosomes, which also exhibit decreased salt stability (171). However, this comparison is difficult to draw even though *S. cerevisiae* H2A can be seen as an orthologue of H2A.X. Slight alterations in amino acid sequences are present in all *S. cerevisiae* histones and distributed throughout the whole nucleosome structure (172), thereby making it hard to evaluate the influence of *S. cerevisiae* H2A on nucleosome stability in an isolated manner. It is tempting to speculate that the extended C-terminal tail present in H2A.X might be involved in the changes discussed earlier in the text, as nucleosome stability is further reduced by C-terminal phosphorylation. Future studies will hopefully reveal which changes in H2A.Xs primary structure are relevant for the observed destabilization.

### H2A.Z

The stability of the H2A.Z-containing nucleosome has been intensively studied with contrasting results [reviewed in (67)]. Some studies found stabilization (86,173,174), whereas others found destabilization of the nucleosome as a consequence of H2A.Z incorporation (78,93,175). Some FRET measurements detected only subtle effects on stability *in vitro* (20,176), consistent with two studies measuring H2A.Z mobility *in vivo* using FRAP (20,177). The reported differences can have a multitude of reasons, for example, the use of H2A.Z from different organisms [note: 80% identity (see earlier in the text) means 20% divergence], different experimental set-ups and different sources of chromatin (recombinant versus native chromatin). Comparison of the available studies is further complicated by the fact that recombinant chromatin consists of homotypic nucleosomes, whereas native chromatin consists of a mixture of homotypic and heterotypic nucleosomes (80–82) that can also be post-translationally modified [Figure 3 and (83)]. Moreover, comparing studies with *in vitro* assembled chromatin is complicated by different DNA sequences used (176). Two examples nicely illustrate these problems; Zhang *et al.* (93) found destabilization by analysing native chromatin fibres prepared from *S. cerevisiae*, whereas Park *et al.* (174) found stabilization by performing FRET analyses of *in vitro* reconstituted nucleosomes from *Xenopus* histones, produced in bacteria, on 5 S rDNA. What one could hypothesize from these studies is that H2A.Z is probably not the sole determinant of nucleosome stability but might modulate it, integrating influences like DNA sequence, PTMs and nucleosome composition.

As mentioned earlier in the text, the crystal structure of the H2A.Z nucleosome revealed differences that might lead to altered nucleosome stability in two regions, namely in L1 and in the docking domain (78). On the one hand, the H2A.Z nucleosome loses hydrogen bonds between docking domain and H3 in comparison with the canonical one, suggesting subtle destabilization. On the other hand, the L1–L1 interface is more extensive by forming more interactions between the dimers in the H2A.Z than in the canonical nucleosome. These additional interactions in the L1 region of the H2A.Z nucleosome led to the proposal that homotypic H2A.Z nucleosomes are more stable, resulting in their enrichment over genes in the fly as discussed earlier in the text (81). One intriguing possibility, which can be envisioned from the crystal structure, is that the (homotypic) H2A.Z nucleosome could be, on the one hand, more stable, because of more extensive interactions in the L1 region, and, on the other hand, could have a more dynamic interface with H3, because of the loss of interactions in this area. If this is true, the H2A.Z nucleosome, compared with the canonical one, could be more resistant towards dimer loss [as discussed in (81)] and could be more susceptible towards the more open nucleosome structure, with all histones bound to DNA but without interactions between dimers and tetramer (12,16). Thus far, however, no rigorous analysis concerning the influence of L1 for H2A.Z nucleosome stability is known; studies focusing on the influence of the H2A.Z C-terminus and docking domain on nucleosome stability are discussed later in the text.

In the past 2 years, the importance of the H2A.Z C-terminus for nucleosome stability and chromatin association has been explored in *S. cerevisiae* and humans (20,21,178). Two studies in *S. cerevisiae* revealed that C-terminal deletions, depending on the extent of truncation, decrease or completely abolish chromatin association (23,178). As expected, loss of chromatin association leads to phenotypes similar to the complete knock-out of the H2A.Z gene in *S. cerevisiae*, such as reduced resistance to genotoxic stress and spreading of heterochromatin into euchromatic regions, indicating that chromatin association is essential for H2A.Z function. Interestingly, by analysing chimeric proteins, both groups found that the C-terminus of canonical H2A can completely restore chromatin association and rescue the H2A.Z knock-out phenotype, consistent with the idea that the primary function of the H2A.Z C-terminus in *S. cerevisiae* is anchoring the protein to chromatin.

The recent discovery of Z.2.2, an alternatively spliced H2A.Z isoform, provided fascinating new insights into the role of H2A.Z's C-terminus (20,21). The novel isoform, Z.2.2, is different from Z.2.1 in two regards as follows: it is 14 amino acids shorter and has a stretch of six unique amino acids in its very C-terminus. We and others have found identical properties with respect to chromatin association and nucleosome stability of Z.2.2 (20,21). This shorter isoform, in contrast to the longer Z.2.1, is not completely associated with chromatin but exhibits a major soluble pool. Moreover, the chromatin-bound fraction is less tightly incorporated into nucleosomes, *in vitro* and *in vivo*, further establishing the importance of H2A.Z's C-terminus in providing stable chromatin incorporation. To further break down which of the two distinguishing properties of Z.2.2's C-terminus, its shortened length or unique amino acid sequence, are critical for its decreased extent and stability of chromatin incorporation, we analysed deletion mutants and chimeric proteins. Surprisingly, mere shortening of Z.2.1 to the same length as Z.2.2 does not dramatically alter chromatin incorporation *in vivo*. In contrast, transferring Z.2.2's unique docking domain to the respective site of H2A results in a protein with chromatin incorporation virtually identical to Z.2.2. These results demonstrate that the specific sequence within Z.2.2's docking domain and not just its shortened length is the critical determinant for the unique properties of Z.2.2 with respect to its incorporation into chromatin. To gain insight into the underlying structural alterations in Z.2.2 nucleosomes, we performed MD simulations that point towards a more flexible C-terminus of Z.2.2, which is also more distant to the H3 αN-helix, thereby reducing interactions with the (H3–H4)_2_-tetramer in a sequence-specific manner. These MD simulations are further supported by Z.2.2's inability to form stable histone octamers under high-salt conditions, which is in contrast to Z.2.1 or canonical H2A. From the results obtained *in silico* and *in vitro*, one can hypothesize that the changed interaction interface, with a striking increase in C-terminal flexibility, leads to less stable DNA organization but increased DNA breathing instead, which is confirmed by decreased resistance to MNase digest. Taken together, Z.2.2 is an intriguing protein that, by specific changes in its C-terminus, drastically alters basic H2A.Z properties possibly leading to a shift in H2A.Z function in certain tissues of high Z.2.2 abundance, for example, brain tissues (20,21).

In line with H2A.Z's role in modulating nucleosome stability as a function of its composition, the Felsenfeld lab reported that H2A.Z severely destabilizes nucleosomes if present with H3.3 in the same particle (179). Nucleosomes prepared from native chromatin containing H2A.Z and H3.3 are highly salt sensitive and are disrupted in the presence of minimal (80 mM) NaCl. In a second article (180), they analysed the genome-wide distribution of H3.3/H2A.Z-containing double variant nucleosomes and found that they mark the NFRs of active promoters, enhancers and insulator regions. These nucleosomes are highly unstable and can, therefore, be more easily replaced by other DNA binding proteins, such as transcription factors. Unfortunately, all experiments used ectopically expressed H3 variants with the tag located at the C-terminus, close to the H3–H3-dimerization interface. Taking into account the dynamic nature of the nucleosome (12,13), this could, potentially, have differential influences on H3.3/H2A.Z-containing double variant nucleosomes compared with those containing H3/H2A.Z *in vivo*. Surprisingly, another study *in vitro* (102), using nucleosomes reconstituted with human histones purified from bacteria, did not find any drastic stability changes for H3.3/H2A.Z-containing double variant nucleosomes. As stated earlier in the text, technical differences in these studies can explain the different outcomes and hamper the drawing of final conclusions.

### H2A.Bbd

Because of its shorter length and highly divergent amino acid sequence [∼50% identical to H2A, Figure 2C and (118)], H2A.Bbd was expected to alter nucleosome structure and organization of DNA significantly. Indeed, several studies investigated H2A.Bbd nucleosome properties mostly using *in vitro* assays, all of which consistently revealed an open structure of H2A.Bbd-containing chromatin. H2A.Bbd organizes DNA less tightly, leading to a more relaxed and elongated structure with ∼180° between the DNA entry/exit sites in contrast to the V-shaped canonical nucleosomes (125,181). These differences in nucleosomal DNA constraint are concomitant with less resistance to digestion by MNase (123,125,181) and a reduced nucleosome repeat length *in vivo* (123). Notably, no H2A.Bbd crystal structure is available thus far, compatible with global structural alterations leading to a more dynamic particle that prevents formation of well-diffracting crystals (12). This is in line with findings from DNaseI footprinting experiments demonstrating significant changes of DNA organization in the H2A.Bbd-containing nucleosome (124,127,181). Analysis of H2A.Bbd nucleosome stability showed that it does not refold into histone octamers under high-salt conditions (20,125), indicating weaker interaction of H2A.Bbd–H2B dimers, with the (H3–H4)_2_-tetramer ultimately resulting in reduced nucleosome stability (20,120,181,182). In accordance with *in vitro* studies discussed earlier in the text, determination of H2A.Bbd mobility *in vivo* using FRAP showed a much faster exchange than canonical H2A (20,183).

Several studies investigated the role of H2A.Bbd's C-terminus for the observed changes in structure and stability. As apparent for Z.2.2, the C-terminus of H2A.Bbd differs from canonical H2A in length and amino acid composition. Hence, the question was whether the shortened length or amino acid sequence is the main determinant for H2A.Bbd's unique properties. Bao *et al.* analysed canonical H2A truncations *in vitro*. They found that mere shortening of the C-terminal tail neither impairs histone octamer assembly under high-salt conditions nor significantly alters DNA organization, indicating that H2A.Bbd's shortened length cannot be the sole determinant for its characteristic properties (125). In contrast, chimeric proteins, consisting of H2A.Bbd's C-terminus/docking domain fused to the N-terminal part of canonical H2A, exhibit properties characteristic for H2A.Bbd. They do not refold into histone octamers under high-salt conditions and bind DNA less tightly with a H2A.Bbd-like geometry, pointing towards an essential role of H2A.Bbd's docking domain in defining interactions with the (H3–H4)_2_-tetramer and DNA (125,127,181). This role is further underlined by the finding that the C-terminus of canonical H2A fused to the H2A.Bbd histone fold is sufficient to organize DNA comparably to canonical H2A nucleosomes and to partly restore the normal V-shaped geometry (181). The analysis of H2AL2, an H2A.Bbd-like protein present in mouse (121,122), revealed striking similarities to human H2A.Bbd, as H2AL2 nucleosomes arrange nucleosomal DNA in a more open structure as canonical ones (184). In conclusion, H2A.Bbd incorporation results in reduction of nucleosome stability and structural constraint of nucleosomal DNA in a manner highly dependent on its docking domain, consistent with its presence at active genes in HeLa cells (123) and during spermatogenesis in mouse (122).

### MacroH2A

The crystal structure of the macroH2A nucleosome provided important clues about alterations on incorporation of this variant (145). Despite the overall high structural similarity to the canonical particle, a four amino acid sequence in L1, which is implicated in interactions between the two H2A–H2B dimers within the nucleosome (Figures 1 and 2), showed noticeable differences. This finding led to the suggestion of increased stability for macroH2A-containing nucleosomes because of stronger interactions between the two macroH2A–H2B dimers. Whether this is indeed the case was addressed by analysis of the macroH2A-containing histone octamer in the absence of DNA. Interestingly, Chakravarthy and Luger (146) found that the macroH2A-containing octamer is less reliant on high-salt stabilization than the canonical one. Canonical octamers dissociate when salt concentration is lowered to 1.1 M NaCl, whereas macroH2A octamers are still completely stable under these conditions. Importantly, by mutational studies, the authors could show that the four amino acid substitutions in the L1 region are solely responsible for the changes observed in octamer stability, pointing towards the importance of the L1 region in defining interactions within the nucleosome. Consistent with these findings, analysis of native chromatin from chicken cells showed an increased stability of macroH2A chromatin incorporation as well (162). Taken together, macroH2A increases nucleosome stability by alterations within a four amino acid stretch in L1, which is in strong contrast to Z.2.2 or H2A.Bbd that lead to a significant decrease in nucleosome stability mediated by their characteristic docking domains.

Further evidence supporting macroH2A's role in constituting nucleosomes that are more stable and static is provided by the finding that chaperone-assisted H2A(variant)–H2B dimer exchange is inhibited by macroH2A-containing nucleosomes (146). Interestingly, the L1 region and the docking domain are not sufficient to transfer this property to canonical H2A, thereby indicating the importance of other regions for macroH2A's static nature. The authors state that the best explanation for these findings is reduction of macroH2A's relative affinity to the chaperone used (yNAP1) compared with canonical H2A. This raises a point neglected in most *in vitro* studies, namely, the influence of the relative affinity of histone variants to factors other than the nucleosome, such as chaperones and remodelling complexes. In principle, the affinity of a histone variant to soluble protein complexes promoting its absence from chromatin must also be considered, as these factors are abundant and contribute significantly to the equilibrium between soluble and chromatin-bound histone variants in the cell. However, it is complicated to exhaustively analyse these protein complexes *in vitro* because of their immense diversity in the living cell. On the other hand, *in vivo* assays, such as FRAP, can provide valuable insights; unfortunately, these data are hard to dissect because of the complexity of the experimental system, that is, the cell. Thorough analyses should, therefore, follow a complementary approach using *in vitro* and *in vivo* analyses to compensate for their inherent technical limitations.

## THE INFLUENCE OF H2A VARIANTS ON CHROMATIN STRUCTURE

The work discussed in the last section focused on the influence of H2A variants on the nucleosome and its basic properties. To understand how H2A variants can alter the ‘monomeric building block’ of chromatin provided us with plenty of insight into the mechanisms of their biological functions. In the cell, however, chromatin is not present in a linear ‘beads-on-a-string’ conformation but adopts higher-order structures impacted by the complex interplay of DNA, core and linker histones and other chromatin architectural proteins [reviewed in (185–188)]. Studies *in vitro* established that short-range (intrafibre/intramolecular) interactions within a linear chromatin strand lead to a more compact secondary structure, the 30 nm fibre. In addition (or instead), long-range (interfibre/intermolecular) interactions between distinct chromatin fibres lead to large oligomeric tertiary complexes. It should be noted that the generality of the 30 nm fibre presence *in vivo* is highly controversial and will be discussed later.

To understand the intricate relationship between histone variants and chromatin function, the influence of chromatin components on secondary and tertiary chromatin structures must be taken into account. In this section, we will discuss the influence of histone variants on chromatin structure with a special focus on the crucial role of the H2A acidic patch.

### The H2A acidic patch is a key regulator of higher-order chromatin structure

H2A and H2B form an obligate dimer under physiological conditions (189); hence, it is the structure of the H2A–H2B dimer that must be considered to be involved in biologically relevant protein–protein interactions (190). Despite the overall basic nature of histones, the nucleosome crystal structure revealed the presence of an acidic patch on the surface of the H2A–H2B dimer, which mainly consists of H2A residues [six of seven amino acids, Figure 2A (8)]. Interestingly, in this structure, the H4 N-terminal tail (K16-N25) contacts the acidic patch on the adjacent nucleosome, and this contact is required for crystallization (8). In addition to the H4 tail, interactions with at least five more non-histone proteins make the acidic patch an important binding site in chromatin with the potential to differentially contribute to diverse biological processes by its alteration as a result of H2A variant incorporation (190). In support of this notion, interleukin-33 interacts with the acidic patch of H2A or H2A.Z, but binding to H2A.Bbd, which lacks an acidic patch, is strongly decreased (191).

The importance of the H4 tail for the establishment of proper secondary and tertiary chromatin structure has been established and depends on its charge and PTMs [reviewed in (185)]. Richmond and co-workers (192) could show that the H4 tail has a critical role beyond the other histone tails for both intra- and interfibre interactions. Furthermore, by using mutant proteins, they could crosslink the H4 tail (H4V21C) to the acidic patch of H2A (H2AE64C, Figure 2A) on array folding, thereby providing evidence for the direct interaction in solution if and when a more compact secondary structure, the 30 nm fibre, is formed (193). More recently, interfibre crosslinks between H4V21C and H2AE64C (Figure 2A) have been reported as well (194). However, additional contacts between chromatin fibres must be also highly important, as arrays containing only (H3–H4)_2_-tetramers can oligomerize just as nucleosomal arrays (195), and binding of the H4 tail to DNA is another important mechanism for the establishment of interfibre interactions (196).

The modulation of chromatin folding by PTMs is of particular importance, as histone PTMs are abundant and implicated in a multitude of biological processes (4). Two H4 tail modifications have been studied *in vitro* with regards to their influence on chromatin folding, acetylation of lysine 16 (197) and trimethylation of lysine 20 (198). These two modifications seem to have opposing biological functions, as H4K16ac is associated with euchromatin and active transcription, whereas H4K20me3 plays a role in heterochromatin formation [reviewed in (199)]. In accordance with these opposing biological functions, H4K16ac inhibits intra- and interfibre interactions of the H4 tail and consequently promotes an open chromatin structure (197). Contrariwise, H4K20me3 leads to more efficient intrafibre folding, resulting in a more compact secondary chromatin structure without influencing interfibre interactions [(198), reviewed in (187)]. Taken together, interaction of the H4 tail with the acidic patch of H2A is important for short-range (intrafibre/intramolecular) and long-range (interfibre/intermolecular) chromatin interactions and can be modulated by PTMs of the H4 tail and incorporation of H2A variants (see later in the text).

### Alterations of the acidic patch by H2A variant incorporation influence higher-order chromatin structure

Two H2A variants, H2A.Z and H2A.Bbd, have been studied with regards to the influence of their acidic patch on secondary and tertiary chromatin structure *in vitro*. These studies contributed significantly to our understanding of the importance of the acidic patch on higher-order chromatin structure. Compared with canonical H2A, H2A.Z has an extended acidic patch, whereas H2A.Bbd virtually lacks it (Figures 2B and C, respectively). In H2A.X and macroH2A, the residues constituting the acidic patch are completely conserved.

**Figure 4.**
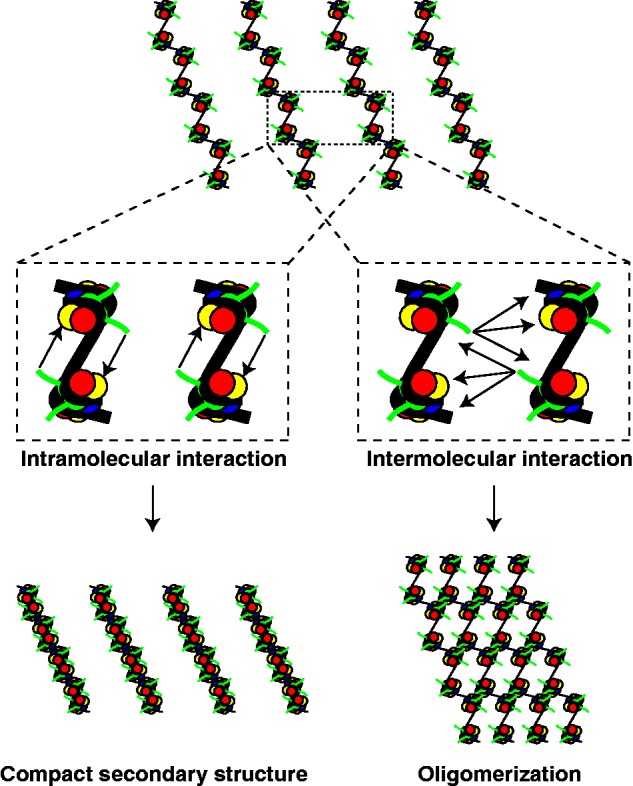
The acidic patch regulates chromatin structure by interaction with the H4 tail. The H4 tail can engage in intrafibre interactions with the acidic patch of neighbouring nucleosomes within the same chromatin fibre to form more compact secondary structures (left). Alternatively, it can form different interfibre interactions with DNA and histones of other chromatin fibres to form large tertiary oligomeric complexes (right). Which interactions are preferred is influenced by acidic patch alterations in H2A variants. The extended acidic patch of H2A.Z fosters compact secondary structure formation (right), whereas the reduced acidic patch of H2A.Bbd leads to preferred oligomerization (left). DNA is shown in black, H2A in yellow, H2B in red, H3 in blue and H4 in green. Flexible histone tails for histones other than H4 are omitted for clarity.

Ten years ago, Tremethick and co-workers (98) investigated the influence of H2A.Z on chromatin folding. They reported that arrays assembled with H2A.Z exhibit increased intrafibre folding and, therefore, a more compact secondary structure than canonical arrays. Interestingly, H2A.Z arrays impair interfibre contacts and, therefore, array oligomerization. Two years later, the authors extended their studies by using acidic patch mutants and H4 tail deletions to mechanistically understand H2A.Z's influence on chromatin folding (200). They found that the extended acidic patch of H2A.Z is responsible for the observed condensed array structures in an H4 tail dependent manner. In this case, the larger acidic patch of H2A.Z has a higher affinity to the H4 tail than H2A allowing stronger electrostatic interactions (200). Furthermore, they demonstrated that the heterochromatin binding protein 1α (HP1α), a functional constituent of heterochromatic regions [for a recent review on HP1 see (201)], recognizes secondary chromatin structures independently of H3K9 methylation, as it preferentially binds highly folded H2A.Z- over H2A-containing chromatin fibres. In addition, HP1α binding further enhances intrafibre folding without bridging chromatin fibres, highlighting the role of histone variants in establishing higher-order chromatin in concert with other structural chromatin proteins. These findings could also be relevant for the establishment and maintenance of centromeric chromatin structure and function *in vivo* as Greaves *et al.* suggested (202). They reported the presence of H2A.Z at major satellite repeats in pericentric heterochromatin and minor satellite repeats in centromere protein A (CENP-A)-containing centric chromatin and proposed that structural changes induced by H2A.Z might be important to compact H3K4me2-containing euchromatin within CENP-A-containing centric chromatin regions. To explain opposing influences of H2A.Z's extended acidic patch on chromatin compaction and oligomerization, they proposed a model based on competition between intra- and interfibre interaction partners for the H4 tail (Figure 4). The H4 tail can either interact with the acidic patch within one fibre, resulting in a more compact secondary structure, or it can participate in other contacts that promote array oligomerization, for example, with DNA of another chromatin fibre (196). Hence, the interaction of the H4 tail with the acidic patch in an intrafibre manner inhibits any other (interfibre) interaction and consequently inhibits oligomerization. Therefore, the stronger the interaction of the acidic patch with the H4 tail, the more favoured the compact secondary structure and the less favoured array oligomerization and *vice versa*.

To test whether the competition model applies more generally, the Tremethick lab analysed arrays assembled *in vitro* with H2A.Bbd, canonical H2A and acidic patch mutants of both (126). In line with the proposed model, H2A.Bbd inhibits intrafibre folding but fosters interfibre oligomerization. Moreover, H2A.Bbd mutants, with a restored acidic patch, increase the tendency to form compact secondary structures depending on the H4 tail and the extent of the acidic patch restoration, whereas mutants of canonical H2A, that lack the acidic patch, form compact secondary structures less efficiently than wild-type but oligomerize chromatin fibres more efficiently. Taken together, these analyses support the competition model and further strengthen the view of the acidic patch as a key regulator of chromatin structure. On the one hand, the extended acidic patch of H2A.Z results in a more efficient formation of compact secondary structures, whereas at the same time it inhibits oligomerization of chromatin fibres. On the other hand, the smaller acidic patch of H2A.Bbd has contrasting effects by fostering oligomerization at expense of secondary structure formation (Figure 4).

The lack of an acidic patch in H2A.Bbd could also explain why no crystal structure is available thus far. Luger *et al.* (8) reported that the contact of the H4 tail with the acidic patch, which cannot be formed with H2A.Bbd nucleosomes, is required for crystallization. Whether this is the only reason for the inability of H2A.Bbd nucleosomes to be crystallized could be easily tested by using the reported H2A.Bbd mutant with a restored acidic patch (126).

Further evidence of how sensitive chromatin folding responds to alterations of the acidic patch was provided by the experimental comparison of human H2A.Bbd with its mouse homologue (122). A single amino acid substitution from human to mouse (T100D) increases the acidic patch of mouse H2A.Bbd (H2A.Lap1) that in turn is able to partially fold chromatin into more compact secondary structures. Mutation of this residue back to threonine, as found in the human protein, disables higher-order chromatin folding, indicating its functional importance. Intriguingly, two of the other H2A.Bbd-like proteins in mouse (H2A.Lap2/3) seem to be more similar to human H2A.Bbd than H2A.Lap1, as they do not have an additional acidic residue that could partially restore the acidic patch (122). This might lead to functional (sub)specialization of the different H2A.Bbd-like proteins in mouse by differential influence on higher-order chromatin structure because of their slightly altered acidic patch.

Interestingly, Z.2.2 combines features of H2A.Bbd and H2A.Z. On the one hand, it significantly destabilizes nucleosomes, similarly to H2A.Bbd; on the other hand, it completely retains the extended acidic patch of H2A.Z (Figure 3). Thus far, no analysis of chromatin folding and oligomerization of Z.2.2 containing arrays are available, but from the literature on the acidic patch, one would expect that Z.2.2, although severely destabilizing nucleosomes, behaves like H2A.Z and allows the formation of compact secondary chromatin structures because of the key role of the acidic patch. This would give Z.2.2 an intriguing role by promoting a compact chromatin structure of unstable nucleosomes. Future studies will ascertain whether this is indeed the case.

In addition to their structural analyses, Zhou *et al.* (126) also functionally investigated the influence of the acidic patch and its impact on secondary and tertiary chromatin structure on transcription. Surprisingly, efficient transcription can occur within large oligomeric chromatin structures and is only impaired by compact secondary chromatin structures formed by H2A.Bbd mutants with a restored acidic patch. This observed structural effect on transcription is consistent with H2A.Bbd's euchromatic localization and its role in gene activation (118,122,123). For H2A.Z, these findings would suggest a repressive role in gene transcription when localized in large chromatin domains; however, at gene promoters, H2A.Z has only been found in few individual nucleosomes (see earlier in the text). It is, therefore, difficult to draw any conclusions about the real occurrence of H2A.Z-dependent compact secondary chromatin structures that prevent transcription in living cells. An interesting exception from this rule might be found in genes important for cell differentiation in mouse ES cells (203). Here, H2A.Z co-localizes strongly with Polycomb repressive complexes (PRCs) 1 and 2 surrounding the TSS of genes, whose expression is important for cell differentiation, in domains of ≥2 kb. Here, together with PRCs and H3K27me3, H2A.Z helps to maintain these genes in a silent state. The authors suggest that in ES cells, in contrast to its function in transcriptional activation, H2A.Z, together with PRC function, sets up a repressive chromatin landscape. Although they do not analyse whether the repressive effect by H2A.Z is mediated through a more compact secondary structure, they find that H2A.Z localization patterns change during differentiation concomitant with a decrease of H2A.Z domain size. Moreover, in differentiated cells, H2A.Z enrichment correlates with increased gene transcription. These findings are consistent with a functional switch of H2A.Z chromatin depending on the size of the H2A.Z enriched domain, ascribing activating function to H2A.Z’s punctuated enrichment at promoters and repressive functions to its enrichment in larger domains, possibly because of a more compact secondary structure for the latter.

### The generality of the 30 nm fibre existence *in vivo* and its implications for acidic patch interactions

As mentioned earlier in the text, many reports indicate that histone variants have marked influences on the generation of higher-order chromatin structures. But how do such structures look like *in vivo*? The significance of the 30 nm fibre has been demonstrated by many groups *in vitro* (185–188), but its general existence *in vivo* is highly debated (204–206). So far, evidence for the 30 nm fibre *in vivo* was only found in chicken erythrocytes, a specialized cell type that is transcriptionally silenced, and in starfish spermatozoids (205,207). However, a recent study on the structure of HeLa mitotic chromosomes did not find any evidence for periodic regular structures >11 nm (beads-on-a-string); hence, no significant cohorts of 30 nm fibres seem to exist in Hela mitotic chromosomes (205). Recently, the idea that chromatin folding, in interphase nuclei and mitotic chromosomes, does not require a 30 nm fibre was put forward (204–206). As Maeshima *et al.* (204) stated, analyses of chromatin *in vitro* can easily lead to artifacts because of non-physiologically low concentrations of nucleosomes, that promote the formation of intrafibre contacts and hence highly regular 30 nm fibres. *In vivo*, however, the nucleosome concentration is much higher and as the nucleosome cannot ‘distinguish’ which nucleosome is within the same fibre and which one is in another one, the formation of interfibre contacts is more likely than the formation of a highly regular 30 nm fibre. Alternatively and in agreement with recent studies (205,208,209), the ‘polymer melt’ model proposes that chromatin fibres in a beads-on-a-string conformation strongly interdigitate and thereby dynamically and irregularly organize chromatin in a fractal manner. However, the existence of short stretches of 30 nm fibres *in vivo* cannot be ruled out (210).

What would the possible absence of the 30 nm fibre mean for the influence of acidic patch alterations on chromatin structure? Interactions of the acidic patch with the H4 tail would still be important. However, according to Maeshima *et al.* and consistent with the reported interfibre crosslinks of H4 tail and H2A acidic patch (194), it would not matter whether the nucleosomes are on the same chromosome fibre. Even if the 30 nm fibre does not exist, and intrafibre contacts are negligible, H2A.Z with its increased acidic patch would lead to stronger interactions with all neighbours (in the same fibre or in other fibres), whereas H2A.Bbd would lead to overall weaker interactions. Consequently, H2A.Z would favour more compact structures, whereas H2A.Bbd would lead to more open ones. The final structural outcome, although different in its molecular details, would not change dramatically.

Another important player involved in the establishment of higher-order chromatin structure is the linker histone H1 (185,187,211) that acts by neutralizing the negative charge of the DNA backbone and, therefore, fosters fibre–fibre interaction and chromatin compaction. As discussed earlier in the text, H2A.Bbd and H2A.Z mononucleosomes bind H1 less efficiently than canonical H2A (102,127). To our knowledge, linker histone binding has not been analysed on H2A variant containing chromatin fibres, which might influence this interaction by generating different secondary and tertiary structures. However, if H2A.Z chromatin indeed binds H1 less efficiently than canonical H2A *in vivo*, this might compensate for the higher tendency of H2A.Z to form compact secondary structures and even out structural differences between H2A- and H2A.Z-containing chromatin. More interestingly, it is tempting to speculate that H2A.Z and H2A form structurally and functionally different chromatin because of their different inherent properties to engage in secondary and tertiary chromatin folding as well as in recruitment of H1 and other chromatin factors.

In conclusion, incorporation of H2A variants into chromatin can alter its secondary and tertiary structure *in vitro*. The key regulators for these alterations are the H2A acidic patch and the H4 tail, which together define different kinds of interactions with distinct structural and functional outcomes. We speculate that, even in light of the emerging view of chromatin organization without regularly folded 30 nm fibres *in vivo*, the implications of acidic patch alterations in H2A variants analysed *in vitro* have meaningful structural outcome *in vivo*. Moreover, the different affinities of H2A variant containing nucleosomes to the linker histone H1 could also play an important role in specifying distinct chromosomal domains. Although a lot of progress has been made in the past decade, the complex composition of chromatin *in vivo* makes it hard to set-up suitable models *in vitro.*

An intriguing additional function of alterations within the acidic patch of histone variants could be a change of their susceptibility to ATP-dependent chromatin remodelling complexes. Goldman *et al.* (212) tested this hypothesis by comparing binding of ATP-dependent chromatin remodellers to canonical and H2A.Z nucleosomes in addition to analysing their activities on different substrates; nucleosomes containing canonical H2A, H2A.Z or mutants of both to analyse the role of the acidic patch. They found that many ATP-dependent chromatin remodelling complexes interact with canonical and H2A.Z nucleosomes to different extents, consistent with the notion of them having different roles on the two kinds of nucleosomes. Furthermore, activity of ISWI containing complexes, but not of other ones, is stimulated by H2A.Z containing nucleosomes, and, at least for the isolated motor protein subunits SNF2H and SNF2L, reliant on the acidic patch. However, H2A.Z's extended acidic patch is not the sole determinant here, as it is required but not sufficient for stimulation of remodelling activity. Moreover, whole complexes, such as ACF, can overcome this requirement. These results suggest that alterations of the acidic patch in H2A variants could confer different properties to chromatin also by more indirect means, not only by influencing chromatin structure and altering interactions with different proteins (see earlier in the text), but also by changing activities of chromatin factors that act on them, like ATP-dependent chromatin remodelling complexes. It is important, however, to note that evidence found by analysing ATP-dependent chromatin remodelling of H2A variant nucleosomes *in vitro* must be complemented with suitable *in vivo* studies, as *in vivo* chromatin’s properties are too complex to be reflected completely by chromatin assembled *in vitro*.

## CONCLUDING REMARKS

The importance of H2A variants in a multitude of biological processes is well established; however, the mechanisms by which they function are not completely understood. From the literature available today, one can envision that histone variants, in general, and H2A variants, in particular, function by conferring characteristic properties to chromatin on the nucleosomal and higher-order structural level. For an overview see Table 1.

**Table 1.** Overview of the influences of H2A variants on nucleosome stability and chromatin structure in comparison with canonical H2A

Histone Variant	Nucleosome stability			Chromatin structure		
	Output	Literature	Relevant domains	Output	Literature	Relevant domains
H2A.X	Destabilization	(49)		ND		
H2A.Z	Controversial:	Review: (67)		Compaction	(98,200)	Acidic patch (extended)
	Stabilization:	(86,173,174)				
	Destabilization:	(78,93,175)				
	No or subtle effects:	(20,176,177)				
H2A.Z.2.2	Destabilization	(20,21)	Docking domain	ND		
H2A.Bbd	Destabilization	(20,120,181–183)	Docking domain	Decompaction	(122,126)	Acidic patch (decreased)
MacroH2A	Stabilization	(145,146,162)	L1 loop	Compaction	(164)	H1-like linker (without macro domain)

ND = not determined.

Our mechanistic understanding of chromatin structure alterations and histone domains involved revealed the functional significance of different regions in H2A, like the L1 loop, the docking domain and C-terminal tail, and the acidic patch. The recent discovery of the H2A.Z splice isoform Z.2.2 (20,21) questions the completeness of our knowledge on existing histone variants.

Although a lot of progress was made in the past decade, we are far from understanding the structure/function interplay of H2A variants. Notably, the importance of H2A variants in a tissue-specific manner was shown for H2A.Bbd (122) and was suggested for Z.2.2 (20,21). In future studies, it will be of particular interest to analyse histone variants with respect to their tissue specific influences on chromatin structure and function.

## FUNDING

German Research foundation (DFG), SFB Transregio 5 and Center for Integrated Protein Science Munich (CIPSM). Funding for open access charge: DFG.

*Conflict of interest statement*. None declared.
